# Reduced Serum Levels of Triglyceride, Very Low Density Lipoprotein Cholesterol and Apolipoprotein B in Parkinson’s Disease Patients

**DOI:** 10.1371/journal.pone.0075743

**Published:** 2013-09-26

**Authors:** Qiang Wei, Honghao Wang, Yanghua Tian, Fangcheng Xu, Xianwen Chen, Kai Wang

**Affiliations:** Department of Neurology, the First Affiliated Hospital of Anhui Medical University, Hefei, Anhui Province, China; University of Iowa Carver College of Medicine, United States of America

## Abstract

**Background:**

Previous studies have shown a lower incidence of stroke in Parkinson’s disease (PD) patients. The role of the lipids and lipoproteins as risk factors for stroke is uncertain in the lower prevalence of stroke in PD patients.

**Objectives:**

To explore the lipids and lipoproteins serum levels in PD patients.

**Methods:**

A retrospective study was performed on 110 PD patients (PD group), 130 controls with non-cerebrovascular neurological diseases (OD group), 140 acute intracerebral hemorrhage patients (ICH group) and 140 acute cerebral infarction patients (CI group). The records about serum levels of lipids and lipoproteins were analyzed.

**Results:**

There were significant differences for the serum level of triglyceride (F = 5.031, *p*=0.002), very low density lipoprotein cholesterol (F = 5.313, *p*=0.001), apolipoprotein B (F = 16.038, *p*<0.0001) in the four groups. PD group had a significantly lower serum level of triglyceride (TG) than the OD (*p*=0.032), ICH (*p*=0.00047) and CI (*p*=0.001) groups. Very low density lipoprotein cholesterol (VLDL-C) serum level was significantly lower in PD group than in OD (*p*=0.039), ICH (*p*=0.00021) and CI (*p*=0.001) groups. There was a significantly lower serum level of apolipoprotein B (apo B) in PD group than in OD (*p*=0.002), ICH (*p*<0.0001) and CI (*p*<0.0001) groups.

**Conclusions:**

There are reduced serum levels of TG, VLDL-C and apo B in PD patients, which may be related to the decreased prevalence of stroke in PD patients.

## Introduction

Parkinson’s disease (PD) is an age-related disorder that classically presents with extrapyramidal symptoms of tremor, rigidity, bradykinesia and postural instability, and includes non-motor symptoms such as depression, anxiety, muscular pain and other disturbances. The occurrence of PD is due to the neuronal degeneration of substantia nigra pars compacta, which causes the deficiency of dopaminergic neurotransmission in the basal ganglia [1,2].

PD significantly lowers the quality life of affected patients. Although not confirmed by some authors [3], epidemiological studies seem to show a reduced incidence of stroke in PD patients. Struck et al [4] and Nataraj and Rajput [5] have found a lower prevalence of stroke in PD patients compared to the general population. In addition, Korten et al have found only eight individuals with PD among 1,516 stroke patients, which are significantly lower than the expected 30 PD patients [6].

However, the reason for the reduced stroke risk in this patient population is uncertain. There are mounting evidences to support that lipids and lipoproteins are closely linked to stroke [7,8]. The modification of lipids and lipoproteins may contribute to the reduced risk of stroke in PD patients. It is known that high blood levels of lipoproteins such as very low density lipoprotein (VLDL) and low density lipoprotein (LDL) transport triglycerides and cholesterols to peripheral blood vessels and accelerate the development of atherosclerosis. It has been shown that total cholesterol (TC), triglyceride (TG), high density lipoprotein cholesterol (HDL-C), very low density lipoprotein cholesterol (VLDL-C), low density lipoprotein cholesterol (LDL-C), apolipoprotein A (apo A), apolipoprotein B (apo B) and other lipids are associated with stroke [7–10]. The modification of TG in PD patients has been reported. Scigliano et al have found that TG were significantly lower in PD patients than in control patients with other non-cerebrovascular neurological disorders [11].

These findings suggest that the modification of TG may be related to the low incidence of stroke in PD patients. It is known that a series of lipids and lipoproteins are associated with stroke. Other lipids and lipoproteins may be also related to the decreased prevalence of stroke in patients with PD. However, more studies are required to clarify the change of lipids and lipoproteins in PD patients.

Here, we performed a retrospective study to explore the serum levels of lipids and lipoproteins in PD patients.

## Methods

All patients were identified retrospectively as inpatients from 2007 to 2013 at the first affiliated hospital of Anhui Medical University. PD patients (PD group) who met the Calne’s criteria of clinically definitive PD [12] were selected consecutively in this study, and patients with atypical degenerative parkinsonisms, secondary parkinsonisms, severe liver disease, severe kidney disease, cancer, vomiting, anorexia, a history of using lipid-regulators or lack of lipids records were excluded. 110 PD patients were included in PD group (male, 62; female, 48; mean age, 67.07±8.74 years). Disease severity according to the Hoehn and Yahr scale [13] was stage I in 30 patients, stage II in 20, stage iii in 44, stage iv in 14, stage Ⅴin 2. Controls with other non-cerebrovascular neurological diseases (OD group), patients with an initial acute intracerebral hemorrhage (ICH group) and patients with an initial acute cerebral infarction (CI group) were chosen by single-blinding method at about the same admitted time in the same hospital. In the process of choosing patients, patients with severe liver disease, severe kidney disease, cancer, vomiting, anorexia, a history of using lipid-regulators, or lack of lipids records were also excluded, and the diagnosis of acute cerebral infarction or acute intracerebral hemorrhage should be confirmed by magnetic resonance imaging (MRI) or computed tomography (CT). Also, for each of the three groups, the age, gender and the history of hypertension, diabetes mellitus, coronary artery disease were matched with PD group. Finally, 130 OD group patients (males, 77; females, 53; mean age, 64.98±10.72 years), 140 ICH group patients (males,83; females, 57; mean age, 64.51±11.60 years) and 140 CI group patients (males, 80; females, 60; mean age, 65.44±11.72 years) were included in this study. The non-cerebrovascular neurological diseases of OD group patients included viral encephalitis, Tolosa-Hunt syndrome, myasthenia gravis, cervical spondylotic myelopathy, lumbar disc herniation, epilepsy and other disorders. The serum levels of TC, TG, HDL-C, VLDL-C, LDL-C, apo A, apo B and lipoprotein a (LP (a)) were determined. Our study was approved by The Anhui Medical University Ethics Committee, and written consents were given by all the patients for their information to be stored in the hospital database and used for research. Anonymized data were used for the statistical analyses.

All the statistical analyses were performed with the SPSS 19.0 for Windows (SPSS, Chicago, IL) and the statistical significance was set at p<0.05. The measurement data were presented as Means ± SD. Chi-square test was used for the comparison of enumeration data. The differences of age among groups were determined with one-way analysis of variance (ANOVA). The univariate general linear model (GLM) was used to test the differences of lipids and lipoproteins between groups holding constant gender, age and the history of hypertension, diabetes mellitus ,coronary artery disease. LSD (least significant difference) method was performed for post-hoc comparisons of all groups based on estimated marginal means.

## Results

The demographics, history of hypertension, diabetes mellitus, coronary artery disease of the four groups were showed in Table **1**. There was no significant difference among groups in age (F = 1.244, *p*= 0.293), gender (χ^2^ = 0.338, *p*=0.953) and history of hypertension (χ^2^ = 1.288, *p*=0.732), diabetes mellitus (χ^2^ = 0.973, *p*=0.808), coronary artery disease (χ^2^ = 1.435, *p*=0.697).

**Table 1:** The demographics, history of coronary artery disease, hypertension, diabetes mellitus of the four groups.

	PD (n=110)	OD (n=130)	ICH (n=140)	CI (n=140)	χ2/F	*p*
Age(year)	67.07±8.74	64.98±10.72	64.51±11.60	65.44±11.72	1.244	0.293
Gender(M/F)	62/48	77/53	83/57	80/60	0.338	0.953
Hypertension(Y/N)	43/67	47/83	60/80	56/84	1.288	0.732
Diabetes mellitus(Y/N)	7/103	8/122	7/133	11/129	0.973	0.808
Coronary artery disease(Y/N)	2/108	2/128	5/135	4/136	1.435	0.697

PD: Parkinson’s disease group; OD: other non-cerebrovascular neurological diseases group; ICH: intracerebral hemorrhage patients group; CI: cerebral infarction patients group; n: numbers; M: male; F: female; Y: yes (ill with this disease); N: no (not ill with this disease); χ^2^: Chi-square test value; F: one-way analysis of variance value.

**Figure 1:**
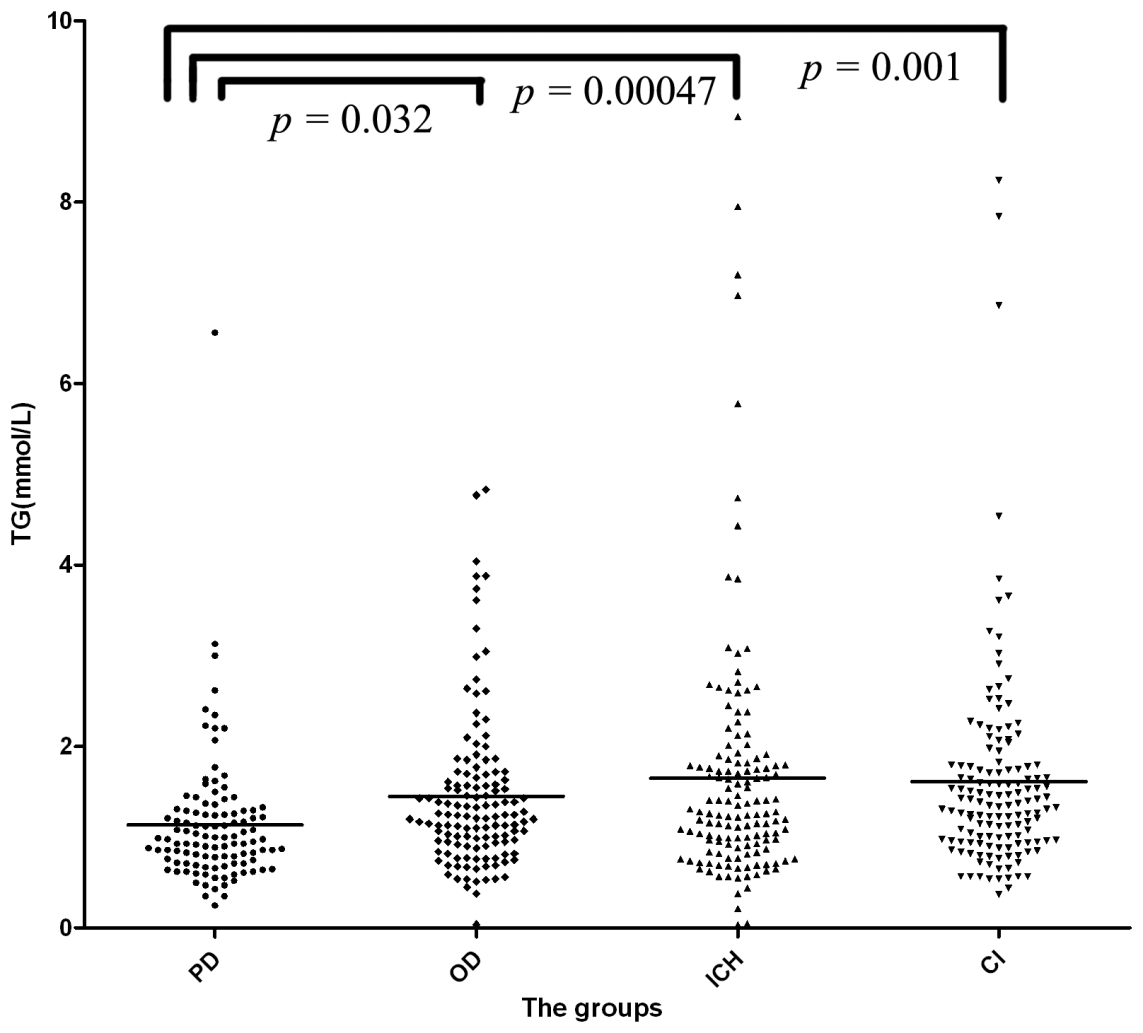
The serum level of triglyceride (TG) in the four groups. PD group had a significantly lower serum level of triglyceride (TG) than the OD (*p*=0.032), ICH (*p*=0.00047) and CI (*p*=0.001) groups. PD: Parkinson’s disease group; OD: other non-cerebrovascular neurological diseases group; ICH: intracerebral hemorrhage patients group; CI: cerebral infarction patients group.

As shown in Table **2**, Table **3** and Table **4**, the serum level of TG was 1.14±0.74 mmol/L in PD group, 1.45±0.86 mmol/L in OD group, 1.65±1.38 mmol/L in ICH group and 1.61±1.16 mmol/L in CI group. The serum levels of TG for the four groups were significantly different (F = 5.031, *p*=0.002). PD patients had a significantly lower serum level of TG than subjects in the OD (*p*=0.032), ICH (*p*=0.00047) and CI groups (*p*=0.001) (Figure **1**). There was no significant difference among the OD, ICH and CI groups.

**Table 2:** The serum levels of lipids and lipoproteins in the four groups.

	PD	OD	ICH	CI
TC(mmol/L)	4.41±0.98	4.58±0.80	4.73±1.01	5.03±1.12
TG (mmol/L)	1.14±0.74	1.45±0.86	1.65±1.38	1.61±1.16
VLDL-C(mmol/L)	0.42±0.28	0.54±0.32	0.63±0.53	0.60±0.43
LDL-C(mmol/L)	2.55±0.83	2.71±0.71	2.69±0.92	3.15±1.08
HDL-C(mmol/L)	1.44±0.40	1.34±0.47	1.47±0.43	1.31±0.39
Apo A (g/L)	1.33±0.31	1.32±0.34	1.42±0.37	1.34±0.32
Apo B (g/L)	0.80±0.21	0.89±0.20	0.92±0.31	1.01±0.25
LP(a) (mg/L)	170.62±171.92	188.04±158.12	191.66±182.79	236.17±201.99

PD: Parkinson’s disease group; OD: other non-cerebrovascular neurological diseases group; ICH: intracerebral hemorrhage patients group; CI: cerebral infarction patients group; TC: total cholesterol; TG: triglyceride; VLDL-C: very low density lipoprotein cholesterol; HDL-C: high density lipoprotein cholesterol; LDL-C: low density lipoprotein cholesterol; Apo A: apolipoprotein A; Apo B: apolipoprotein B; LP(a): lipoprotein a.

**Table 3:** The results of univariate general linear model for lipids and lipoproteins in the four groups.

	TC	TG	HDL-C	VLDL-C	LDL-C	Apo A	Apo B	Lp(a)
F	9.342	5.031	4.449	5.313	12.428	2.721	16.038	3.095
*p*	<0.0001*	0.002*	0.004*	0.001*	<0.0001*	0.044*	<0.0001*	0.027*

The results were adjusted for gender, age and the history of hypertension, diabetes mellitus ,coronary artery disease using univariate general linear model.

* p< 0.05; PD: TC: total cholesterol; TG: triglyceride; VLDL-C: very low density lipoprotein cholesterol; HDL-C: high density lipoprotein cholesterol; LDL-C: low density lipoprotein cholesterol; apo A: Apolipoprotein A; Apo B: apolipoprotein B; LP(a): lipoprotein a;F: univariate general linear model value.

**Table 4:** The post-hoc comparisons of lipids and lipoproteins in the four groups.

	**TC**	**TG**	**HDL-C**	**VLDL-C**	**LDL-C**	**APOA**	**APOB**	**LP(a)**
**PD VS OD**	0.142	0.032*	0.099	0.039*	0.147	0.773	0.002*	0.438
**PD VS ICH**	0.011*	0.00047*	0.380	0.00021*	0.221	0.030*	<0.0001*	0.388
**PD VS CI**	<0.0001*	0.001*	0.032*	0.001*	<0.0001*	0.766	<0.0001*	0.005*
**OD VS ICH**	0.270	0.162	0.008*	0.088	0.795	0.010*	0.351	0.937
**OD VS CI**	0.00022*	0.244	0.623	0.251	<0.0001*	0.536	0.00012*	0.033*
**ICH VS CI**	0.008*	0.809	0.001*	0.568	<0.0001*	0.046*	0.003*	0.036*

The results were adjusted for gender, age and the history of hypertension, diabetes mellitus ,coronary artery disease using univariate general linear model.

* p< 0.05; PD: Parkinson’s disease group; OD: other non-cerebrovascular neurological diseases group; ICH: intracerebral hemorrhage patients group; CI: cerebral infarction patients group; TC: total cholesterol; TG: triglyceride; VLDL-C: very low density lipoprotein cholesterol; HDL-C: high density lipoprotein cholesterol; LDL-C: low density lipoprotein cholesterol; Apo A: apolipoprotein A; Apo B: apolipoprotein B; LP(a): lipoprotein a.

**Figure 2:**
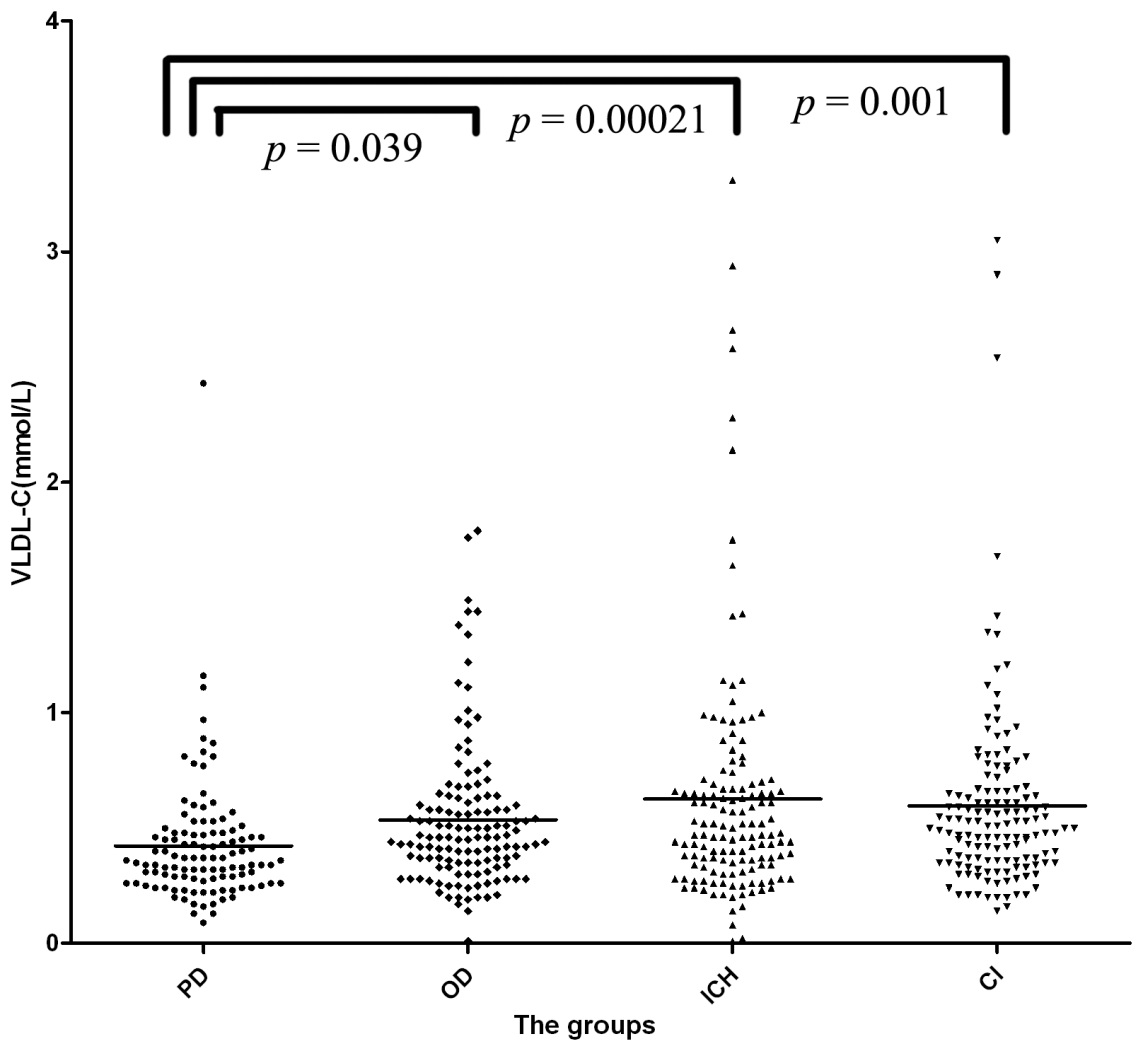
The serum level of Very low density lipoprotein cholesterol (VLDL-C) in the four groups. Very low density lipoprotein cholesterol (VLDL-C) serum level was significantly lower in PD group than in OD (*p*=0.039), ICH (*p*=0.00021) and CI (*p*=0.001) groups. PD: Parkinson’s disease group; OD: other non-cerebrovascular neurological diseases group; ICH: intracerebral hemorrhage patients group; CI: cerebral infarction patients group.

The VLDL-C serum level was 0.42±0.28 mmol/L in PD group, 0.54±0.32 mmol/L in OD group, 0.63±0.53 mmol/L in ICH group and 0.60±0.43 mmol/L in CI group. The serum levels of VLDL-C for the four groups were significantly different (F =5.313, *p*=0.001). The serum VLDL-C level was significantly lower in PD group than in the OD (*p*=0.039), ICH (*p*=0.00021) and CI groups (*p*=0.001) (Figure **2**). However, no significant difference was observed among the OD, ICH and CI groups.

**Figure 3:**
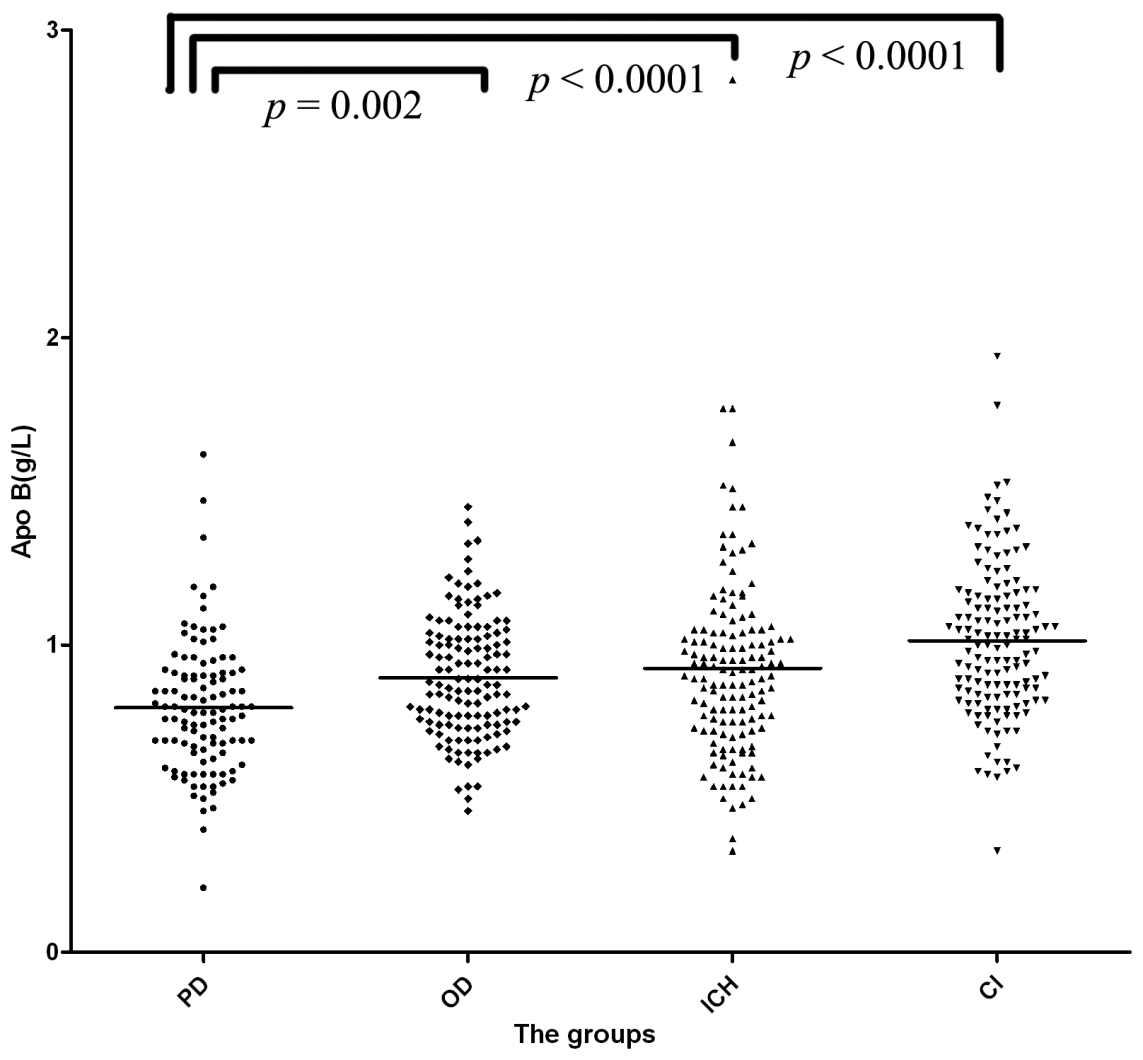
The serum level of apolipoprotein B (apo B) in the four groups. There was a significantly lower serum level of apolipoprotein B (apo B) in PD group than in OD (*p*=0.002), ICH (*p*<0.0001) and CI (*p*<0.0001) groups. PD: Parkinson’s disease group; OD: other non-cerebrovascular neurological diseases group; ICH: intracerebral hemorrhage patients group; CI: cerebral infarction patients group.

The serum level of apo B was 0.80±0.21 g/L in PD group, 0.89±0.20 g/L in OD group, 0.92±0.31 g/L in ICH group and 1.01±0.25 g/L in CI group. The serum levels of apo B were significantly different (F = 16.038, *p*<0.0001) in the four groups. There was a significantly lower serum level of apo B in the PD group than in OD group (*p*=0.002), ICH group (*p*<0.0001) and CI group (*p*<0.0001) (Figure **3**). Serum levels of apo B were lower in the OD group than in ICH group and CI group, but the difference was only significant when compared to the CI group (*p*=0.00012).

The serum level of TC was 4.41±0.98 mmol/L in PD group, 4.58±0.80 mmol/L in OD group, 4.73±1.01 mmol/L in ICH group and 5.03±1.12 mmol/L in CI group. The serum levels of TC were significantly different (F = 9.342, *p*<0.0001) among the four groups. The TC serum level was significantly lower in PD group than in CI group (*p*<0.0001) and in the ICH group (*p*=0.011). But there was no significant difference between the PD group and OD group(*p*=0.142).

The serum LDL-C level was 2.55±0.83 mmol/L in PD group, 2.71±0.71 mmol/L in OD group, 2.69±0.92 mmol/L in ICH group and 3.15±1.08 mmol/L in the CI group. The serum levels of TC for the four groups were significantly different (F =12.428, *p*<0.0001). The LDL-C serum levels were significantly lower in PD group than in CI group(*p*<0.0001), but there was no significant difference when the LDL-C serum level of the PD group was compared to OD group (*p*=0.147) and ICH group (*p*=0.221).

The serum level of HDL-C was 1.44±0.40 mmol/L in PD group, 1.34±0.47 mmol/L in OD group, 1.47±0.43 mmol/L in ICH group and 1.31±0.39 mmol/L in CI group. A significantly difference was observed for the HDL-C serum levels of the four groups(F =4.449, *p*=0.004). But no significant difference of the serum level of HDL-C was observed when the PD group compared with OD group (*p*=0.099), ICH group (*p*=0.380). The HDL-C serum level was significantly higher in PD group than in CI group (*p*=0.032).

The serum level of apo A was 1.33±0.31 g/L in PD group, 1.32±0.34 g/L in the OD group, 1.42±0.37 g/L in ICH group and 1.34±0.32 g/L in CI group. The serum levels of apo A for the four groups were not significantly different (F =2.721, *p*=0.044). The apo A serum level was significantly lower in PD group than in ICH group (*p*=0.030), but not in OD group (*p*=0.773) and in CI group (*p*=0.776).

The serum level of LP (a) was 170.62±171.92 mg/L in PD group, 188.04±158.12 mg/L in OD, 191.66±182.79 mg/L in ICH and 236.17±201.99 mg/L in the CI groups. There was significantly difference for the LP (a) serum levels of the four groups(F =3.095, *p*=0.027). The serum level of LP (a) was significantly lower in PD group than in CI group (*p*=0.005), but no significant difference was observed when PD group compared with OD group (*p*=0.438) and ICH group (*p*=0.388).

## Discussion

High plasma levels of lipids and lipoproteins and low levels of HDL-cholesterol are established risk factors for stroke [7,8]. Our goal was to find the lipids and lipoproteins that may contribute to the reduced incidence of stroke in PD patients. In this study, we have adjusted the age ,gender and the history of hypertension, diabetes mellitus ,coronary artery disease among the four groups to avoid the biases of these confounding factors. Finally, we find that the serum levels of TG, VLDL-C and apo B are the most remarkable indexes that are significantly lower in PD group than in OD, CI and ICH groups.

The possible relationship between serum TG level and stroke has been investigated in various studies. In a systematic review, Labreuche et al have found a positive association between TG and stroke [14]. Many researchers have also performed case control studies to investigate the level of TG in stroke patients compared with other non-cerebrovascular diseases patients or health control, and TG has been identified as a risk factor for stroke [15-17]. Scigliano et al also found low level of plasma TG in PD patients which was consistent with our study [11]. Taken together, these data suggest that decreased risk of stroke in PD patients could be related to low level of TG in these patients.

VLDL is an independent risk factor for atherosclerosis and has been associated with the incidence of stroke [18,19]. VLDL transport triglyceride (VLDL-T) and cholesterol (VLDL-C) from the liver to the target tissues, including peripheral blood vessels. One study has shown that vaccination with VLDL to induce neonatal tolerance can protect endothelial cells and reduce the accumulation of lipids in rats [20]. It has been reported that VLDL-C is significantly associated with the thickness of the intima media of the common carotid artery, which reflects the degree of arteriostenosis [21]. In case control studies, significantly higher levels of VLDL-C have also been found in stroke patients than in health controls [10,22]. In our study, the serum level of VLDL-C is significantly lower in the PD group than the other three groups. These results indicate that lower VLDL-C may contribute to the reduced stroke risk in PD patients.

Apo B is a main structural protein of the VLDL, LDL and chylomicrones. The apo B-containing lipoproteins transport lipids from the liver and intestine to the sites of utilization, such as blood vessels. When the apo B-containing lipoproteins reach the endothelial and smooth muscles of blood vessels, the process of arteriosclerosis which is an important risk factor of stroke is initiated [23-25]. It has been demonstrated that high levels of apo B-containing lipoproteins such as VLDL and LDL promote the development of arteriosclerosis [26]. The apo B level is the representation of apo B-containing lipoproteins, i.e., a higher level of apo B indicates higher levels of VLDL and LDL. These transport more TC and TG to the blood vessels and lead to more severe arteriosclerosis. Epidemiological studies have already demonstrated this and shown that increased levels of apo B is a risk factor of stroke [9,27]. In our study, the serum level of apo B was significantly lower in the PD group. These results suggest that a lower level of apo B may also contribute to the decreased prevalence of stroke in PD patients.

As the core protein of VLDL, a reduced serum level of apo B is consistent with the reduced serum level of VLDL-C in PD patients. The majority of TG is transported by VLDL, which is rich of TG. The reduced serum level of TG indicates a reduced serum level of VLDL, which leads to a reduced serum level of apo B. We found that the serum levels of all the substances were significantly lower in the PD patients than the control subjects, which is consistent with the close relationship among them. According to the discussion above, we hypothesize that the synergetic effect of reduced TG, VLDL-C and apo B in serum leads to a lower incidence and a slower progression of arteriosclerosis, which results in a lower prevalence of stroke in PD patients.

Nutrition contributes to the lipid profile. Van der Marck et al have concluded that PD patients have a significantly lower body mass index (BMI) than controls in a meta-analysis [28], but Barichella et al have reported that nutrition is generally normal in PD patients [29]. So it needs further research to explore the relationship between nutrition and the changes of lipids and lipoproteins in PD patients.

Scigliano et al have suggested that these changes may be related to the reduced sympathetic activity that characterizes PD [11]. In that study, authors have found TG levels in PD patients have significantly lower than in the control group which is consistent with reduced catecholamine, cortisol production. But Berg et al have found that the serum concentration of apo B-containing lipoproteins could not be regulated by cortisol and catecholamines, and adrenocorticotrophic hormone (ACTH) could reduce the serum concentration of apo B-containing lipoproteins [30]. One study has reported that ACTH is significantly lower in untreated PD patients than in health control group [31]. According to above studies, there may be a higher serum concentration of apo B-containing lipoproteins in PD patients. But the study is performed on untreated PD patients, which could not represent the real state in treated PD patients, especially the effect of levodopa. Levodopa can be converted into dopamine by enzymes in peripheral tissues, and one study has reported that dopamine could decrease serum TG level [32]. Consistently, Scigliano et al have already found that there is a significantly lower plasma levels of TG and total lipids in levodopa-treated patients than in untreated patients which may be the peripheral effects of levodopa [33]. These evidences suggest that levodopa may play a key role in the changes of lipids and lipoproteins in PD patients.

For further research, it is important to confirm that levodopa could regulate lipids and lipoproteins in PD patients. Well-designed randomized self controlled studies need to be performed which aim to clarify the changes of lipids and lipoproteins in PD patients before and after treatment. On the basis, researchers should pay more attention to molecular mechanisms of lipids and lipoproteins regulated by levodopa, which may extend to new lipids regulatory mechanism and effective lipid regulators. Also, nutrition, as a controversial role in the metabolism of lipids and lipoproteins in PD patient, need more studies to identify the exact role.

## Conclusions

There are reduced serum levels of TG, VLDL-C and apo B in PD patients, which may be related to the decreased prevalence of stroke in PD patients.
